# Use of Automated Machine Learning for Classifying Hemoperitoneum on Ultrasonographic Images of Morrison’s Pouch: A Multicenter Retrospective Study

**DOI:** 10.3390/jcm12124043

**Published:** 2023-06-14

**Authors:** Dongkil Jeong, Wonjoon Jeong, Ji Han Lee, Sin-Youl Park

**Affiliations:** 1 Department of Emergency Medicine, College of Medicine, Soonchunhyang University, Cheonan 31151, Republic of Korea; 397012@naver.com; 2 Department of Emergency Medicine, School of Medicine, Chungnam National University, Daejeon 35015, Republic of Korea; gardenjun@hanmail.net; 3 Division of Emergency Medicine, Department of Medicine, The Catholic University of Korea, Seoul 11765, Republic of Korea; 4 Department of Emergency Medicine, College of Medicine, Yeungnam University, Daegu 42415, Republic of Korea

**Keywords:** ultrasonography, automated machine learning, emergency medicine, trauma, hemoperitoneum

## Abstract

This study evaluated automated machine learning (AutoML) in classifying the presence or absence of hemoperitoneum in ultrasonography (USG) images of Morrison’s pouch. In this multicenter, retrospective study, 864 trauma patients from trauma and emergency medical centers in South Korea were included. In all, 2200 USG images (1100 hemoperitoneum and 1100 normal) were collected. Of these, 1800 images were used for training and 200 were used for the internal validation of AutoML. External validation was performed using 100 hemoperitoneum images and 100 normal images collected separately from a trauma center that were not included in the training and internal validation sets. Google’s open-source AutoML was used to train the algorithm in classifying hemoperitoneum in USG images, followed by internal and external validation. In the internal validation, the sensitivity, specificity, and area under the receiver operating characteristic (AUROC) curve were 95%, 99%, and 0.97, respectively. In the external validation, the sensitivity, specificity, and AUROC were 94%, 99%, and 0.97, respectively. The performances of AutoML in the internal and external validation were not statistically different (*p* = 0.78). A publicly available, general-purpose AutoML can accurately classify the presence or absence of hemoperitoneum in USG images of the Morrison’s pouch of real-world trauma patients.

## 1. Introduction

Uncontrolled massive bleeding is a leading cause of potentially preventable death in major trauma [1]. Massive bleeding from an organ and vascular injury in the chest or abdomen are associated with approximately 20–40% of trauma deaths that occur after hospital arrival, and the early recognition and control of the bleeding can reduce these mortality rates [2,3]. Therefore, the early recognition of massive bleeding in major trauma patients and the rapid control of bleeding while initiating resuscitation are critical steps in the emergency department [4].

Computed tomography (CT) is an imaging modality that is considered the gold standard for assessing hemothorax and hemoperitoneum and the presence of internal organ and vascular injury; however, CT can be difficult to obtain in hemodynamically unstable patients because it requires transport of the patient from the emergency department [5].

Focused assessment with sonography for trauma (FAST) is currently the initial assessment tool of choice for hemodynamically unstable trauma patients and has the advantage of being a simple, non-invasive method with reasonable sensitivity, specificity, and accuracy that can be performed safely, quickly, and repeatedly at the bedside [6,7].

However, the disadvantages of ultrasonography (USG), such as the low clarity of USG images, interobserver variability of the findings, and low quality of acquired images depending on the scanning skill of the operator, can lead to the poor interpretation accuracy of the USG images if the operator is not sufficiently trained in USG or has limited experience in performing it, and poor sensitivity in detecting hemoperitoneum on USG images, especially when the amount of hemoperitoneum is insignificant [8,9,10]. The accuracy of FAST hemoperitoneum diagnosis varies widely, from 61.3% to 100%, depending on the operator [11,12,13].

In the field of medical imaging, deep learning (DL) algorithms can not only reduce the workload of pre-screening but also mark areas of suspected lesions, thereby increasing the reliability of clinicians’ diagnosis from medical images and reducing the likelihood of diagnostic errors [14,15]. In the field of emergency medicine, where clinical decisions must be made within minutes of arrival at the emergency department for resuscitation and stabilization, the help of DL algorithms in classifying these medical images is highly anticipated [16,17].

Recent studies of DL algorithms in radiography and CT have shown that preliminary classification by DL algorithms can help clinicians improve the accuracy of imaging diagnoses [18,19,20]. Based on this observation, we hypothesized that if a DL algorithm can provide accurate medical assistance in classifying hemoperitoneum on trauma USG, it can improve the reliability of hemoperitoneum diagnosis for clinicians who are less experienced in interpreting trauma findings on USG and reduce errors to improve clinical decision making.

Building DL algorithms in USG imaging requires high-performance computing equipment and collaboration with DL coding experts to develop non-sparse DL algorithms, which is a major barrier to DL research by clinicians without the support of equipment and technology [21,22]. Recently, global information technology companies have offered cloud-computing-based automated machine learning (AutoML) platforms that can perform DL without the need for high-performance computing equipment and specialized programming skills. If these AutoML platforms can be applied to medical imaging, the equipment and technical barriers that clinicians face in applying DL algorithms to clinical research can be reduced. As a preliminary investigation into the application of AutoML in classifying hemoperitoneum in trauma USG, this study aimed to evaluate the performance of AutoML in classifying the presence or absence of hemoperitoneum in Morrison’s pouch USG images of trauma patients.

## 2. Materials and Methods

### 2.1. Study Design and Setting

We retrospectively collected USG images of adult trauma patients from multiple medical centers in the Republic of Korea to build and evaluate an AutoML system capable of classifying the presence or absence of hemoperitoneum on USG images of Morrison’s pouch.

### 2.2. Study Population

For the development cohort, adult trauma patients who presented to one level I trauma center, two level I emergency medical centers, and one level II emergency medical center between 1 June 2019 and 31 July 2021, and who initially underwent trauma USG were included in the analysis. Patients who did not undergo USG imaging, whose USG images of Morrison’s pouch were not available, and those who were younger than 18 years were excluded.

### 2.3. Data Collection

**Figure 1:**
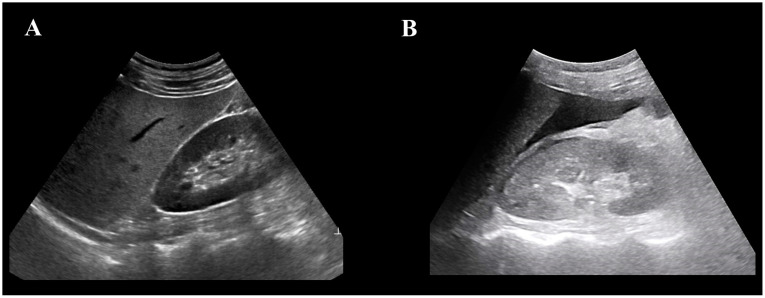
Ultrasonographic image of Morrison’s pouch. (**A**) Normal ultrasonographic image of Morrison’s pouch. Morrison’s pouch is observed as hyperechoic lines. (**B**) Ultrasonographic image with hemoperitoneum in Morrison’s pouch. Hypoechoic fluid collection in Morrison’s pouch can be observed.

An emergency medicine (EM) specialist with at least 10 years of experience performing trauma USG and at least 5 years of experience teaching trauma USG at USG-related academic organizations collected USG images at his emergency medical center using a photo archiving and communication system (PACS). Among the various trauma USG images, only Morrison’s pouch USG images were included in this study. Morrison’s pouch refers to the hepatorenal recess, which is the space between the liver and the right kidney; only USG images in which this area was clearly visible were selected. If hypoechoic blood was observed in Morrison’s pouch, it was classified as hemoperitoneum, and if only hyperechoic lines were observed, it was classified as normal (Figure 1).

The selected USG images were collected as JPG files at a size of 1024 × 768 pixels. All USG images were anonymized and sent to the lead researcher, who first checked the quality and anonymity of the USG images.

The lead researcher checked the anonymity, clarity, and size of the USG images collected from each hospital. Then, after randomly shuffling the collected USG images so that the classification of normal and hemoperitoneum could not be confirmed, three separate emergency medicine experts with more than 10 years of FAST clinical experience were recruited and asked to reclassify the presence or absence of hemoperitoneum in the Morrison’s pouch USG images. If there was disagreement on the classification of the presence or absence of hematoperitoneum, the classification was determined by consensus among the three experts.

### 2.4. USG Image Distribution

A total of 2200 USG images from 864 patients were collected from one level 1 trauma center, two level 1 urgent care centers, and one level 2 urgent care center.

Training and internal validation of the AutoML were performed with a total of 2000 USG images (1000 hemoperitoneum USG images and 1000 normal Morrison’s pouch USG images) collected from 782 patients at the three emergency medical centers, of which 1800 randomly selected images (900 hemoperitoneum USG images and 900 normal USG images) were used for training the AutoML. The remaining 200 images (100 hemoperitoneum USG images and 100 normal USG images) were used for internal validation.

**Figure 2:**
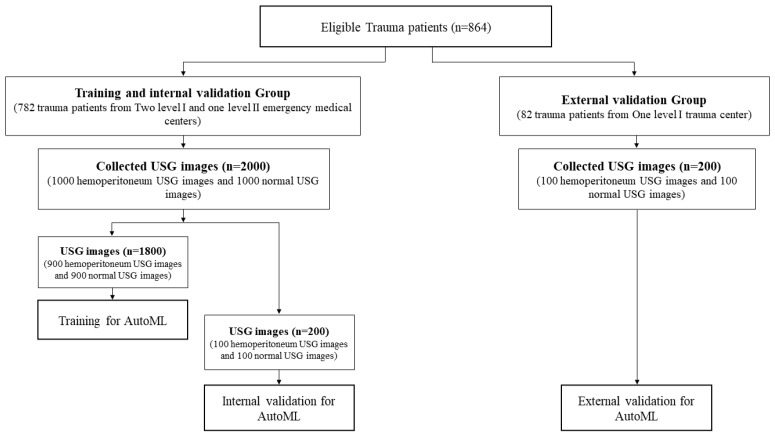
Distribution of ultrasonography images from eligible trauma patients for training, internal validation, and external validation of automated machine learning.

External validation of AutoML was performed using 200 USG images (100 hemoperitoneum images and 100 normal images) collected separately from a Level 1 trauma center that was not included in the training and internal validation. The training, internal validation, and external validation of AutoML and the distribution of USG images are shown in Figure 2.

### 2.5. USG Image Analysis with AutoML

**Figure 3:**
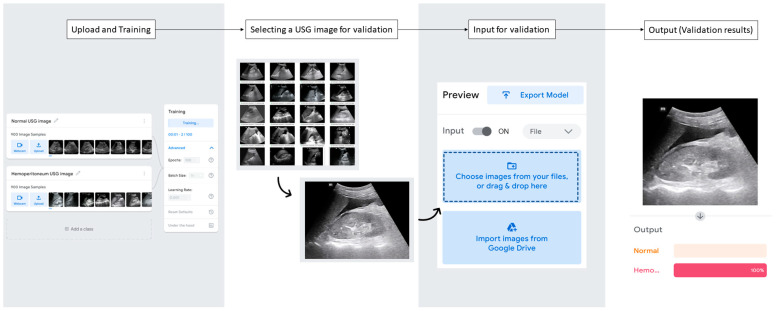
Training and validation of automated machine learning.

To train the AutoML for classifying the presence or absence of hemoperitoneum in trauma USG images, we used the open-source DL provided by Google (https://teachablemachine.withgoogle.co, accessed on 1 March 2023) (Figure 3).

AutoML, popularized this time, is an online platform that can classify images, actions, sounds, and more. The professional work involved in DL coding is already built in, so users can train AutoML by simply uploading images or videos by class after deciding which class to classify. Detailed training settings, such as epoch, batch size, and training speed, can be selected by the user. By clicking the “Training” button, the custom DL is trained; training takes a few minutes depending on the training settings and the number of images. Once the AutoML is trained, users can validate it by uploading individual images or videos to the “Input” section and see the results in real time in the “Output” section of the AutoML. The results in the “Output” section are displayed as the size of the color bar graph and the percentage of the class to which the video and image you uploaded for validation belong.

The training and validation process of AutoML for classifying the presence or absence of hemoperitoneum in trauma USG images in this study is shown in Figure 3. Two classes, “normal” and “hemoperitoneum”, were created for training AutoML in this study. For training, 900 normal USG images were dragged and dropped into the “normal” class, and similarly, 900 hemoperitoneum USG images were dragged and dropped into the “hemoperitoneum” class. The training settings used 100 epochs, batch size of 16, and learning rate of 0.001. Training took approximately 1 min.

Then, the trained AutoML was validated using 100 normal USG images and 100 hemoperitoneum USG images. After randomly mixing the 200 USG images prepared for internal validation, the USG images were dragged and dropped one at a time into the validation input field, and the results were checked and recorded. In the “Output” section, the groups represented by the size and percentage of the color bar graph were defined as “AutoML’s Classification”. If both groups were represented in the color bar graph, the group with the higher percentage was defined as “AutoML’s Classification”. The results of the 200 internal validations were compared to the classifications of an emergency physician with 10 years of experience, categorized as negative and positive, and a confusion matrix based on the results was created.

External validation of AutoML was performed using 200 separately collected USG images that were not used in the training and internal validation. After randomly mixing 100 normal USG images and 100 hemoperitoneum images, we uploaded the USG images one at a time by dragging and dropping them into the validation “Input” section and recorded the results. We defined the AutoML classification results in the same way as the internal validation, and the results were compared with the standard reference results to create a confusion matrix.

### 2.6. Statistical Analysis

The Shapiro–Wilk test was used to test for normality of the continuous variables. When the data did not follow normal distribution, medians (95% confidence interval [CI]) were used, and the Mann–Whitney U test was performed to test for significance. Categorical variables were expressed as frequency (percentage), and statistical significance was assessed using either the chi-squared or Fisher’s exact test. The discriminatory performance of AutoML in recognizing hemoperitoneum in Morrison’s pouch during internal and external validation was evaluated in terms of sensitivity, specificity, positive predictive values (PPV), and negative predictive values (NPV). The performance of AutoML in both validation groups was compared with that of three EM experts using the McNemar test for paired proportions. The statistical differences between the internal and external validation groups in terms of sensitivity, specificity, PPV, and NPV were evaluated using the two-proportions test. The performance of the AutoML was evaluated by comparing the area under the receiver operating characteristic (AUROC) curves of the internal and external validation groups. Statistical tests were conducted using MedCalc (version 19.4.1, MedCalc Software, Ostend, Belgium), and all tests were two-sided, with *p*-values considered significant at 0.05.

## 3. Results

### 3.1. Clinical Characteristics of Enrolled Trauma Patients

A total of 2200 USG images were collected from USG still frames and video clips of 864 patients. Of these, 2000 USG images were collected from 782 patients at three hospitals and were used for training (1800 images) and internal validation (200 images). The remaining 200 USG images were separately collected from 82 patients at one hospital and were used for external validation. The sample population included 596 men (68.98%) and 268 women (31.01%), with a median age of 58 years (95% CI, 56.0–60.0). Of the enrolled patients, 429 (49.65%) had hemoperitoneum. Demographic data of the patients in the training and internal and external validation groups are presented in Table 1; no statistically significant differences were found between the two groups.

### 3.2. Performance of AutoML in Classifying Hemoperitoneum in Morrison’s Pouch USG Images: The Internal Validation Group

Table 2 shows the internal validation results of AutoML classifying the presence or absence of hemoperitoneum in USG images of Morrison’s pouch. The performance of AutoML in the internal validation showed a sensitivity of 95% (95% CI, 88.72–98.36%), a specificity of 99% (95% CI, 94.55–99.97%), a PPV of 98.96% (95% CI, 93.11–99.85%), and an NPV of 95.19% (95% CI, 89.39–97.90%). The accuracy was 97.00% (93.58–98.89%).

When comparing the internal validation results of AutoML with the classification results of the three EM experts, the statistical difference was found to be 2.00% (95% CI, −0.38–4.38%). There was no statistically significant difference between the performance of the EM experts and the AutoML in classifying the presence or absence of hemoperitoneum in the USG images of Morrison’s pouch (*p* = 0.22).

### 3.3. Performance of AutoML in Classifying Hemoperitoneum in Morrison’s Pouch USG Images: The External Validation Group

In the external validation, the performance of AutoML showed a sensitivity of 94% (87.40–97.77%), a specificity of 99% (95% CI, 94.55–99.97%), a PPV of 98.95% (95% CI, 93.04–99.85%), an NPV of 94.29% (95% CI, 88.36–97.29%), and an accuracy of 96.50% (92.92–98.58%) (Table 3). The statistical difference between the performance of AutoML in the external validation and the classification by EM experts was 2.50% (95% CI, −0.07–5.07%). There was no statistically significant difference between the performance of the EM experts and the AutoML in classifying the presence or absence of hemoperitoneum in USG images (*p* = 0.13).

### 3.4. Comparison of Internal and External Validation of the Performance of AutoML in Classifying the Presence or Absence of Hemoperitoneum on USG Images of Morrison’s Pouch

Table 4 shows the results of a statistical comparison of the internal and external validation of the performance of AutoML in classifying the presence or absence of hemoperitoneum on USG images of Morrison’s pouch. The difference in the performances between internal and external validation indicated a sensitivity of 1% (95% CI, −3.70–5.76%), a specificity of 0% (95% CI, −2.67–2.67%), a PPV of 0.01% (95% CI, −2.69–2.71%), and an NPV of 0.90% (95% CI, −3.72–5.58%). There were no statistically significant differences in the performance of the two groups in terms of sensitivity, specificity, PPV, and NPV.

### 3.5. ROC Curve of AutoML in Classifying the Presence or Absence of Hemoperitoneum in Morrison’s Pouch USG Images

**Figure 4:**
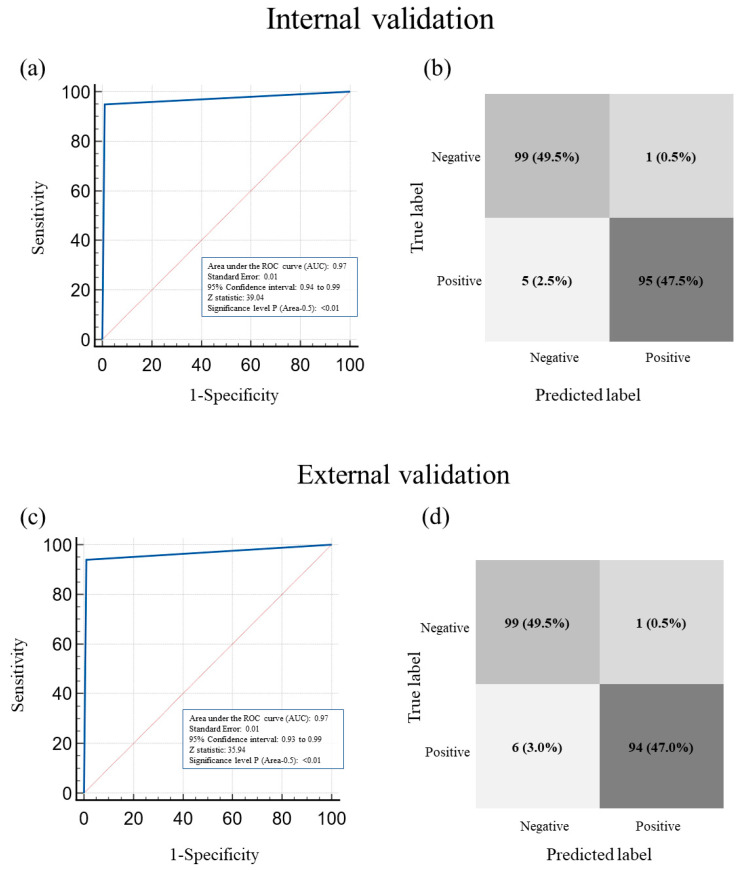
Receiver operating characteristic (ROC) curves and confusion matrix for the performance of AutoML in classifying the presence or absence of hemoperitoneum in Morrison’s pouch USG image. (**a**) ROC curve of automated ML classification of hemoperitoneum in Morrison’s pouch USG image in internal validation. (**b**) Confusion matrix of AutoML classification of hemoperitoneum in Morrison’s pouch USG image in internal validation. (**c**) ROC curve of AutoML classification of hemoperitoneum in Morrison’s pouch USG image in external validation. (**d**) Confusion matrix of AutoML classification of hemoperitoneum in Morrison’s pouch USG image in external validation. The statistical difference in the AUROC between the internal and external validation groups was 0.01 (standard error, 0.02), and the difference in the AUROC between the two groups was not statistically significant (*p* = 0.78). USG, ultrasonography; and AutoML, automated machine learning.

Figure 4 shows the confusion matrix and ROC curve for the performance of AutoML in classifying hemoperitoneum in USG images in the internal and external validation groups, respectively. The AUROC values for the internal and external validation groups were 0.97 (95% CI, 0.94–0.99) and 0.97 (95% CI, 0.93–0.99), respectively. The statistical difference in AUROC values between the internal and external validation groups was 0.01 (standard error, 0.02), and there was no statistically significant difference between the two groups (*p* = 0.78).

## 4. Discussion

The purpose of this study was to evaluate the performance of AutoML in classifying the presence or absence of hemoperitoneum in USG images of Morrison’s pouch using the AutoML platform. To our knowledge, this is the first study to evaluate the performance of a publicly available, general-purpose AutoML platform that requires no technical support or equipment to classify USG images of real-world trauma patients.

Independent external validation with separate USG images showed that a publicly available, general-purpose AutoML can classify the presence or absence of hemoperitoneum in Morrison’s pouch USG images of real trauma patients with a high accuracy and performance comparable to that of USG-trained emergency physicians.

The utility of USG in the primary and secondary examination in trauma patients, allowing early bedside interpretation of results in real time while making informed medical decisions about treatment and care, is well-recognized. However, for untrained or inexperienced novice USG users, obtaining good-quality USG images can be challenging, and the accuracy or speed of determining the presence or absence of hemoperitoneum from acquired images can be compromised. This can negatively impact medical decision making for trauma patients.

Recently, DL, one of the most widely used artificial intelligence technologies in medical imaging, has been shown to be useful in various medical fields. In particular, with regard to radiography and computed tomography, DL algorithms have shown a high accuracy in classifying the presence or absence of lesions, comparable to that of related specialists, and DL algorithms can contribute to improving the diagnostic performance of physicians, especially novice physicians, by providing accurate medical assistance [23,24,25,26,27].

In a study by Jin et al., an artificial intelligence solution using chest radiographs showed acceptable diagnostic accuracy in a respiratory outpatient clinic, and the use of the solution improved the diagnostic performance of physicians in judging chest radiographs [28]. Although research on DL with USG images has been less active than that with radiographs and CT and MR images, recent improvements in USG technology and image quality have led to attempts to classify lesions using DL algorithms with a variety of USG images.

Raimondo et al. found that in the diagnosis of adenomyosis using USG images, DL models had a low accuracy but high specificity of diagnosis compared to that of residents with intermediate skills (fourth-year residents), which demonstrated that DL algorithms can be useful in assisting novice physicians with clinical decision making [29]. In addition, a DL algorithm built to support lesion detection in breast USG images was able to discriminate between benign and malignant breast nodules with a diagnostic accuracy equivalent to that of experts, demonstrating that DL algorithms can classify the presence or absence of lesions in USG images [30].

Furthermore, Sjogren et al. examined the use of DL algorithms in the field of trauma USG; they evaluated the performance of ML in determining the presence or absence of hemoperitoneum in 20 USG images in the perihepatic view of trauma patients [31]. They found that ML could distinguish between the presence and absence of hemoperitoneum with a sensitivity of 100% and specificity of 90% and concluded that such DL algorithms can help clinicians interpret trauma USG results. A study applying a customized DL algorithm to USG still images and video clips of pediatric trauma patients showed that, compared to experts, the DL algorithm was able to detect hemoperitoneum in Morrison’s pouch USG images with a sensitivity of 91.9% (95% CI, 91.7–92.1%) and a specificity of 97.9% (95% CI, 97.8–98.0%) [32]. The authors showed that the accurate classification of USG images via DL algorithms is critical for ensuring the quality and feasibility of multi-level DL FAST models and improving the assessment of injured children.

Recently, in a study performed to detect hemoperitoneum in Morrison’s pouch on USG images using a bespoke DL model, the model was able to detect the presence or absence of hemoperitoneum in Morrison’s pouch with a sensitivity and specificity of 96.7% and 98.5%, respectively; hence, a DL algorithm with an automated feedback and training system could help improve the reading and image acquisition skills of inexperienced sonographers [33]. Unlike previous studies that used custom-built ML or DL algorithms for trauma USG images, this study evaluated the performance of the algorithm in classifying USG images of real trauma patients using the public AutoML platform, which does not require professional coding or large-scale equipment. We found that AutoML could classify the presence or absence of hemoperitoneum with a high accuracy; its results were comparable to the classification made by relevant experts and consistent with the results of previous studies using bespoke ML and DL algorithms.

The use of AutoML in classifying the presence of hemoperitoneum in USG images of Morrison’s pouch is expected to help improve the accuracy of the assessment by healthcare providers who are unfamiliar or inexperienced in examining or reading trauma USG images.

However, to apply AutoML to real-world clinical patients, it must be convenient in addition to being accurate. This is especially true for USG, which lacks the clarity of other medical imaging modalities, and USG images tend to vary in quality, depending on the skills of the operator. Therefore, for the practical clinical application of AutoML, it is important that AutoML demonstrates high accuracy in classifying not only selected USG images with high clarity in a defined area but also trauma USG images with varying clarity levels in different areas. The clinical value of AutoML as a potential decision support tool needs to be evaluated in a large, multi-center, prospective study of trauma USG images of varying clarity levels at different sites and in different practice models. In addition, to receive real-time medical advice using AutoML-based point of care technologies, technological advances that allow AutoML to be embedded in the USG machine or integrated with a PACS or electronic medical record system are required. However, the advice of AutoML is not perfect; in a real-world clinical setting, clinicians should not rely solely on the advice of AutoML but should consider it in conjunction with other tests and results to make a final medical decision [34]. Nevertheless, medical advice of AutoML is expected to help clinicians improve their clinical decisions when identifying hemoperitoneum on trauma USG images.

### Limitations

Given the retrospective nature of this study, the possibility of bias cannot be excluded. This study also had the following limitations:

First, per the FAST protocol, the left and right upper and lower abdomen are scanned consecutively, and then the images are evaluated collectively to assess the presence or absence of hemoperitoneum. While Morrison’s pouch is a highly specific area for recognizing peritoneum, USG images of this organ alone are not sufficient to assess the presence or absence of hemoperitoneum [35,36]. Because this study was conducted with images of one of three sites within the peritoneal cavity imaged under FAST, the results of this study cannot be directly compared to the performance of FAST by healthcare providers. The results of this study are also limited by the fact that AutoML could only distinguish between the presence and absence of hemoperitoneum on USG images of Morrison’s pouch. Further studies are needed to evaluate the classification performance of AutoML using USG images of the lower and left upper abdomen.

Second, although this study was conducted with USG images of real trauma patients, only high-quality images of Morrison’s pouch with high clarity were selected. It is necessary to evaluate the presence or absence of hemoperitoneum on USG images with varying levels of clarity in clinical practice. Therefore, further AutoML studies involving USG images with varying levels of clarity are necessary.

Third, in this study, we used still-frame images. As the concept of “point-of-care USG” is gaining popularity, free fluid in clinical situations is being identified via a comprehensive evaluation of continuous dynamic images rather than by the evaluation of still images. Additionally, the performance of customized DL algorithms in classifying USG images may be better with video clips than with still frames [32]. Therefore, further research on the performance of AutoML in analyzing USG images from video clips is needed.

## 5. Conclusions

A publicly available, general-purpose AutoML was able to accurately classify the presence or absence of hemoperitoneum in Morrison’s pouch USG images of real-world trauma patients. The assistance provided by AutoML in classifying hemoperitoneum in Morrison’s pouch USG images is expected to improve the accuracy and speed of hemoperitoneum diagnosis using trauma USG images by clinicians unfamiliar or inexperienced in examining and reading trauma USG images.

## Figures and Tables

**Table 1:** Clinical characteristics of enrolled trauma patients.

	Entire Sample (n = 864)	Training and Internal Validation Group (n = 782)	External Validation Group (n = 82)	*p*
Sex (n, %)				0.17
Male	596 (68.98)	534 (68.27)	62 (75.61)	
Female	268 (31.02)	248 (31.71)	20 (24.39)	
Median age (yr) (95% CI *)	58.0 (56.0–60.0)	58 (56.0–60.0)	57 (51.7–62.3)	0.81
Hemoperitoneum (n, %)	429 (49.65)	388 (49.62)	41 (50.00)	0.95

* CI: confidence interval.

**Table 2:** Classification performance of AutoML in classifying hemoperitoneum in Morrison’s pouch USG images in the internal validation group.

	Internal Validation Group	Difference (95% CI) with Standard Reference *	*p*
Sensitivity	95.00% (88.72–98.36%)	2.00% (−0.38–4.38%)	0.22
Specificity	99.00% (94.55–99.97%)		
PPV	98.96% (93.11–99.85%)		
NPV	95.19% (89.39–97.90%)		
Accuracy	97.00% (93.58–98.89%)		

AutoML: Automated machine learning; CI: confidence interval; PPV: positive predictive value; and NPV: negative predictive value. * Standard reference: decision of three emergency medicine experts.

**Table 3:** Classification performance of AutoML in classifying hemoperitoneum in Morrison’s pouch ultrasonography images in the external validation group.

	External Validation Group	Difference (95% CI) with Standard Reference *	*p*
Sensitivity	94.00% (87.40–97.77%)	2.50% (−0.07–5.07%)	0.13
Specificity	99.00% (94.55–99.97%)		
PPV	98.95% (93.04–99.85%)		
NPV	94.29% (88.36–97.29%)		
Accuracy	96.50% (92.92–98.58%)		

AutoML: Automated machine learning; CI: confidence interval; PPV: positive predictive value; and NPV: negative predictive value. * Standard reference: decision of three emergency medicine experts.

**Table 4:** Comparison of the performance of AutoML in internal and external validation in classifying hemoperitoneum on ultrasonographic images of Morrison’s pouch.

	Difference (95% CI) between Internal and External Validation	*p*
Sensitivity	1% (−3.70–5.76%)	0.66
Specificity	0% (−2.67–2.67%)	1.0
PPV	0.01% (−2.69–2.71%)	0.99
NPV	0.90% (−3.72–5.58%)	0.69

AutoML: Automated machine learning; CI: confidence interval. PPV: positive predictive value; and NPV: negative predictive value.

## Data Availability

The data used in this study are not publicly accessible due to image management policy issues at each hospital but are available from the corresponding author upon reasonable request.
